# Differences in PFAS exposure between Pacific pinnipeds: The Galapagos (*Zalophus wollebaeki*) and California (*Zalophus californianus*) sea lions

**DOI:** 10.1016/j.marpolbul.2026.119666

**Published:** 2026-04-01

**Authors:** Ashley E. Cave, Isabella G. Livingston, Taylor M. Gregory, Eleanor C. Hawkins, Shelly Vaden, Marjorie Riofrío-Lazo, Diane Deresienski, Gregory A. Lewbart, Anthony J. Orr, Emily Griffith, Christopher Fuller, Mark J. Strynar, James McCord, Jacqueline Bangma, Alissa C. Deming, Diego Páez-Rosas, Matthew Breen

**Affiliations:** a Department of Molecular Biomedical Sciences, North Carolina State University, Raleigh, North Carolina, 27607, United States; b Department of Clinical Sciences, North Carolina State University, Raleigh, North Carolina, 27607, United States; c Greensboro Science Center, Greensboro, North Carolina, 27455, United States; d Galapagos Science Center, Universidad San Francisco de Quito, Isla San Cristóbal, 200150, Islas Galapagos, Ecuador; e Marine Mammal Laboratory, Alaska Fisheries Science Center, National Marine Fisheries Service, National Oceanic and Atmospheric Administration, Seattle, Washington, 98115, United States; f Department of Statistics, North Carolina State University, Raleigh, North Carolina, 27695, United States; g U.S. Environmental Protection Agency, Office of Applied Science and Environmental Solutions, Research Triangle Park, North Carolina, 27711, United States; h U.S. Environmental Protection Agency, Office of Water, Research Triangle Park, North Carolina, 27711, United States; i The Pacific Marine Mammal Center, Laguna Beach, California, 92651, United States; j Fundación Conservando Galapagos, Galapagos Conservancy, Isla Santa Cruz, 200350, Islas Galapagos, Ecuador; k Dirección del Parque Nacional Galapagos, Unidad Técnica Operativa San Cristóbal, Isla San Cristóbal, 200150, Islas Galapagos, Ecuador; l Comparative Medicine Institute, North Carolina State University, Raleigh, North Carolina, 27607, United States; m Center for Human Health and the Environment, North Carolina State University, Raleigh, North Carolina, 27695, United States

**Keywords:** Per- and polyfluoroalkyl substances (PFAS), Marine pollution, Endangered species, Galapagos sea lion (*Zalophus wollebaeki*), California sea lion (*Zalophus californianus*)

## Abstract

Per- and polyfluoroalkyl substances (PFAS) are a class of man-made persistent chemicals that have been detected in both terrestrial and aquatic environments and thus impact humans, household pets, agricultural species, and wildlife. PFAS exposure is linked to a variety of adverse health consequences, including cancer and immune disruption. This study investigates differences in PFAS exposure between California (CSL, *n* = 69) and Galapagos (GSL, *n* = 65) sea lion pups and juveniles across various sampling locations within each species’ habitat range. Whereas the GSL were all considered healthy, 17 of the 69 CSL were classified as malnourished. Comparing the two species, significantly higher serum concentrations of summed PFAS, perfluorooctanesulfonic acid (PFOS), perfluorononanoic acid (PFNA), and perfluorodecanoic acid (PFDA) were detected in CSL than in GSL. This elevated PFAS exposure in CSL is likely related to historic pollution in their environment and close proximity to the urbanized coastline of Southern California. Within species, malnourished CSL had significantly higher PFNA than healthy CSL pups. GSL pups also tended to have higher PFAS levels than GSL juveniles. Within the Galapagos archipelago, PFAS exposure differed between rookeries, with PFOS exceeding its method reporting limit (0.5 ng/mL) in every sample, including GSL from islands uninhabited by humans. Summed PFAS, PFNA, PFDA, and perfluoroundecanoic acid (PFUnA) were all significantly higher in sea lions sampled on uninhabited than inhabited islands in the Galapagos archipelago. This study is the first comprehensive report of PFAS in CSL and the first archipelago-wide assessment of PFAS in GSL.

## 1. Introduction

Pollution and industrialization threaten the health of humans, animals, and the environment. Global changes in chemical use are reflected in the health of aquatic ecosystems, and marine organisms can serve as valuable sources of data (Bossart, 2011; Evich et al., 2022; Khan et al., 2023). As apex predators, marine mammals are sentinels for the physiological disruption associated with contaminant exposure in the world’s oceans (Bossart, 2011; Krucik et al., 2023; Wittmaack et al., 2015). Population studies of numerous marine mammal species over the years have revealed associations between adverse health outcomes and exposure to persistent organic chemicals (POCs), including cancer (Daniel Martineau et al., 1999; Gulland et al., n.d.), immunotoxicity (Ross et al., 1995; Desforges et al., 2016), endocrine disruption (Mos et al., 2010), and reproductive effects (DeLong et al., 1973; Hall et al., 2006). Elevated exposures to heavy metals (which may occur naturally or anthropogenically) have also been documented in marine mammals, and are also associated with negative health effects, including immune disruption (Desforges et al., 2016). For example, excessive mercury and cadmium levels have been detected in scats of Juan Fernandez fur seals, *Arctocephalus philippii*, despite this species being non-migratory and living in an isolated area in Chile where very few people and no polluting industries are present (Toro-Valdivieso et al., 2023). These contaminants have been associated with a suite of negative health consequences in people and animals (Straif et al., 2009).

Per- and polyfluoroalkyl substances (PFAS) are another major class of chemicals of growing concern that have been detected in a variety of aquatic and marine mammals (Houde et al., 2005; Tao et al., 2006; Gui et al., 2019; Lopez-Berenguer et al., 2020; Kannan et al., 2001; Shaw et al., 2009; Taylor et al., 2021; Sait et al., 2023; Kannan et al., 2006). PFAS are a group of nearly 15,000 synthetic chemicals used broadly in numerous commercial, industrial, and consumer products, leading to their presence in carpets, building materials, waterproof clothing, cosmetics, food packaging, cookware, and firefighting foams (Zahm et al., 2024). The persistent nature of most PFAS means that they remain in the environment and in the body for many years, hence them being referred to as ‘forever chemicals’. Evidence indicates that the biological action of these chemicals is consistent with key characteristics of carcinogens, including oxidative stress, immunosuppression, epigenetic modification, and cell proliferation (Temkin et al., 2020). This is reflected in human and animal studies, where PFAS exposure has been associated with a variety of adverse health outcomes, including cancers (Winquist et al., 2023; Steenland and Winquist, 2021; van Gerwen et al., 2023; Fenton et al., 2021). Perfluorooctanoic acid (PFOA) and perfluorooctane sulfonate (PFOS) are the most abundant and widely studied PFAS. The International Agency for Research on Cancer (IARC) has classified PFOA as a Group 1 carcinogen (“carcinogenic to humans”) and PFOS as a Group 2 carcinogen (“possibly carcinogenic to humans”) (Zahm et al., 2024).

PFAS are not just persistent on land; they are also considered ubiquitous in global marine environments (Khan et al., 2023). PFOA and PFOS, in particular, have been associated with immune disruption in a variety of species, including marine mammals (Kannan et al., 2006; Soloff et al., 2017). When comparing 143 reports of PFAS concentrations in surface water using variable methods from the Arctic, North Atlantic, South Atlantic, North Pacific, South Pacific, Indian, and Southern Oceans, the North Pacific shows the greatest variety of compounds and the second-highest summed PFAS concentrations, in contrast with reports from the Southern Pacific, which have the second-lowest summed PFAS concentrations (Khan et al., 2023).

The Galapagos sea lion [GSL] (*Zalophus wollebaeki)*, endemic to the Galapagos archipelago in the equatorial Pacific, is an endangered species whose population has declined by 50% in just the past 40 years (Páez-Rosas et al., 2021). El Niño events (resulting in poor prey availability), introduced diseases, and pollution, are the main contributing factors to this decline (Trillmich, 2015; Gregory et al., 2023; Denkinger et al., 2017). Exposure to environmental contaminants is known to impair the general health and immune function of marine mammals and may further exacerbate the impacts of these stressors (Ross et al., 1995; Desforges et al., 2016). A recent report of 14 GSL juveniles from the El Malecon rookery in the Galapagos has reported PFAS exposure in this species (Mehdi et al., 2025). Persistent organic pollutants (Alava et al., 2009), dichloro-diphenyl-trichloroethane (DDT) (Alava et al., 2011), oil (Salazar, 2003), plastics (Muñoz-Pérez et al., 2023), and heavy metals (i.e., aluminum, mercury, and lead) (Garcia-Garin et al., 2024; Borrell et al., 2023; Calle et al., 2022) have also been detected in GSL.

Although not endangered, its congener in the North Pacific, the California sea lion [CSL] (*Zalophus californianus*), faces its own health challenges and has the highest incidence of cancer among marine mammals (Gulland et al., 1996; Deming et al., 2018). Neoplasia was the cause of death in 18.5% of CSL necropsied at The Marine Mammal Center (Sausalito, CA) between 1979 and 1994, and 85% of these cases involved a highly aggressive and metastatic urogenital (UG) carcinoma (Gulland et al., 1996). Similar rates of UG carcinoma continued to be detected at the same center between 2005 and 2015, with 14% of the sea lions reported to have metastatic cancer, of which 90% were UG carcinoma (Deming et al., 2018). The waters off of Southern California are polluted with persistent organic pollutants, including the insecticide DDT from the dumping of industrial waste (Young et al., 1976; Xia, 2024). Elevated exposure to persistent contaminants, along with an otariine herpesvirus 1 (OtHV1) infection, are positively associated with UG carcinoma incidence in CSL (Gulland et al., n.d.). PFAS have also been detected in mussels and sediments off the urban coastline of California, including the Southern Bight where the present study takes place (Swam et al., 2024). To our knowledge, the only prior assessment of PFAS in CSL investigated only PFOS in the livers of six adult sea lions in 2001 (Kannan et al., 2001). This coastline is home to various military sites, airports, wastewater treatment plants, and industrial facilities, all of which are major contributors of PFAS into the environment (Salvatore et al., 2022). This includes multiple military sites in the Channel Islands where CSL give birth to their pups (Salvatore et al., 2022).

It is likely that exposure to immunosuppressive contaminants may contribute to disease development and adverse health outcomes in marine mammals (Desforges et al., 2016). However, PFAS exposure has not been comprehensively assessed in CSL and has only recently been reported in a limited number of GSL from one rookery in the Galapagos archipelago (Mehdi et al., 2025). This study provides initial data for PFAS exposure in CSL and GSL across multiple islands in the Galapagos through the detection of serum concentrations of 24 of the most common PFAS.

## 2. Materials and methods

### 2.1. Sample acquisition

**Fig. 1.**
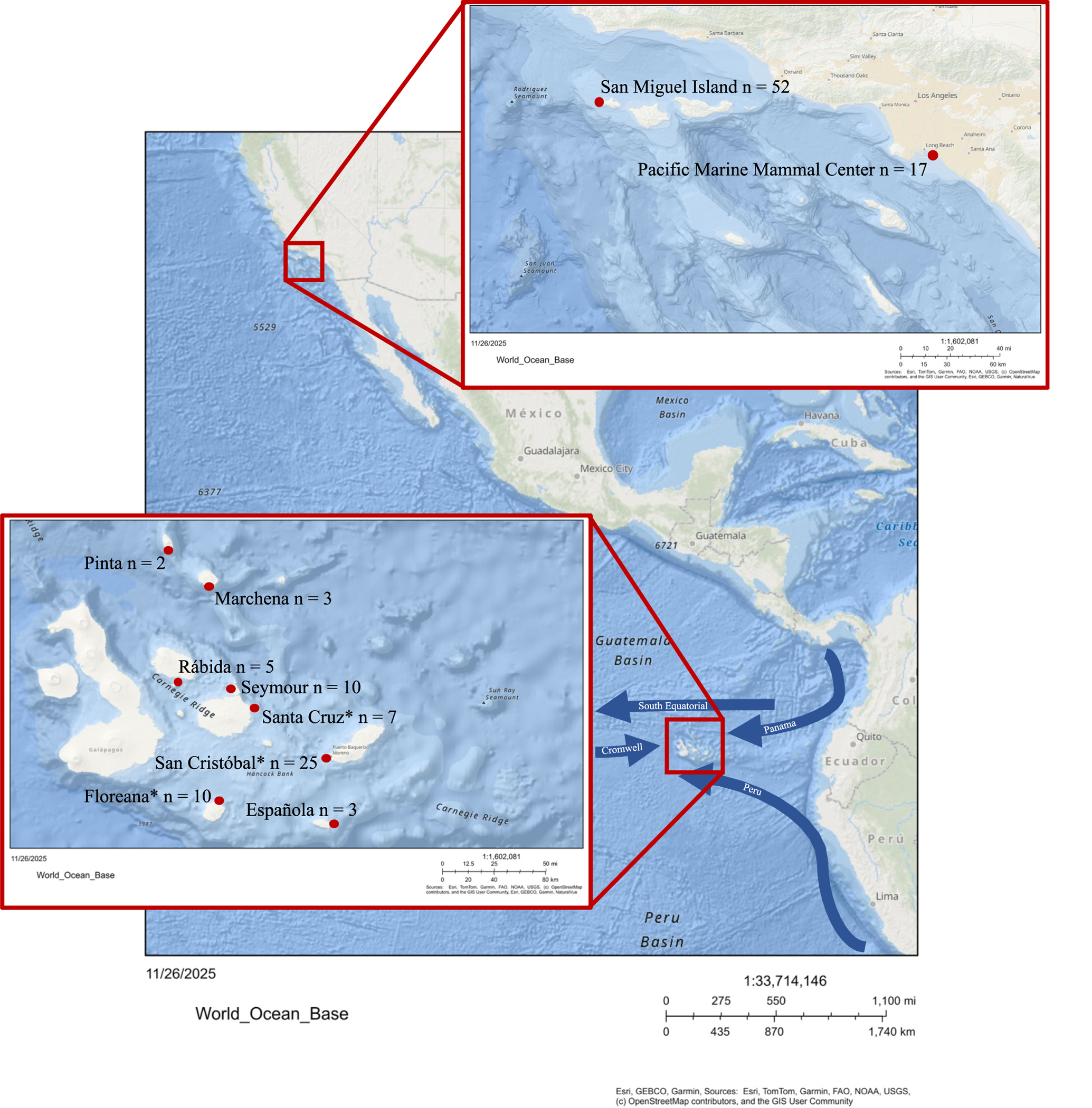
Sampling locations of Galapagos (*Zalophus wollebaeki*) and California (*Zalophus californianus*) sea lions included in this study. Major ocean currents coming into the Galapagos archipelago are represented by blue arrows.

Between May 2022–August 2024, serum samples were collected from pups and juveniles, totaling 134 sea lions (65 GSL and 69 CSL; Table 1). For the GSL, samples were comprised of 25 serum samples from healthy juveniles located at the El Malecón rookery on San Cristobal Island collected in 2022, and 40 serum samples from healthy pups collected in 2024 from Pinta (*n* = 2), Marchena (*n* = 3), Rábida (*n* = 5), Seymour (*n* = 10), Santa Cruz (*n* = 7), Floreana (n = 10), and Española (n = 3) (Fig. 1). Whole blood was collected from GSL, and the samples were transferred under the ethical review and approval granted by the Ministry of the Environment of Ecuador under the Framework Contract for Access to Genetic Resources MAE-DNB-CM-2016–0041 and the research permit DPNG-PC-6–22, PC-19–23, and PC-12–24. All fieldwork was carried out following the protocols of ethics and animal handling approved by the San Francisco de Quito University.

CSL samples were comprised of 52 serum samples from healthy pups from San Miguel Island (11 in 2022, 17 in 2023, 24 in 2024) and 17 serum samples from pups from the California coast admitted to the Pacific Marine Mammal Center, Laguna Beach, CA for rehabilitation for malnutrition from 2022 to 2023. All rehabilitation activities were conducted under a stranding agreement between NOAA’s Marine Mammal Health and Stranding Response Program (MMHSRP) and the Pacific Marine Mammal Center. The 40 healthy GSL pups (average age of six mos) and 24 of the healthy CSL pups (average age of nine mos) were collected within 14 days of each other (Table 1).

For field sampling, sea lions were captured by net and manually restrained. A physical examination was performed by a veterinarian to assess the overall health status of each animal. For healthy CSL pups from San Miguel Island, each pup received isoflurane gas anesthesia and oxygen through a vaporizer with a mask to facilitate handling as part of a population and health assessment project (NMFS Research Permit 22678). Blood was then collected from the caudal gluteal vessel into a serum separator tube, placed on ice, and processed within 6 h of collection.

Last, stranded malnourished CSL (14 pups, three yearlings) received a physical examination by the attending veterinarian within 48 h of arriving at the rehabilitation hospital. Blood was collected under manual restraint from the caudal gluteal vessel into a serum separator tube and processed within 30 min of collection.

Serum samples were aliquoted and placed at − 80 °C for long-term storage until analyzed. In addition, morphometric data including total length (nose-tail length), weight, dentition, sex, and body condition score were collected.

### 2.2. Laboratory analysis

The serfum concentrations of 24 common PFAS were assessed using liquid chromatography high-resolution mass spectrometry (LC-HRMS). Standards were obtained from Wellington Laboratories (Guelph, ON) as native and mass labeled mixes (PFAC24-PAR and MPFAC24ES, respectively). Full PFAS names, abbreviations, and matched internal standards can be found in Supplemental Table 1. For extraction, 100 μL of sea lion serum was added to a 2 mL polypropylene tube, to which 200 μL of 0.1 M formic acid (99.5%, P/N A117, Fisher Scientific) solution containing 5 ng/mL of each internal standard was added; the tube was vortexed for 30 s and allowed to sit for 90 min. Proteins were precipitated by adding 1 mL of −20 °C acetonitrile (Optima^®^, Lot 184,819, Fisher Scientific, Hampton, NC), followed by vortexing for 30 s and centrifugation at 10,000 x*g* for five minutes. 200 μL of the resulting supernatant were added to a polypropylene autosampler vial containing 200 μL of a mobile phase gradient Solvent A (95:5 H_2_O:acetonitrile, 2.5 mM ammonium acetate (>97%, P/N 238074, Sigma Aldrich) and vortexed for 30 s to mix.

Double blanks were prepared with a mixture of the mobile phase Solvent A and the extraction solvent (1:5 formic acid: acetonitrile). The matrix matched blank was created using charcoal stripped fetal bovine serum (Life Technologies, Grand Island, NY; cat #10437, Lot #1754113; total protein 3.7 g/dL) extracted following the same protocol as the samples. Fetal bovine serum was spiked with native PFAS mix to generate a 14-point, matrix-matched calibration curve (0.1–150 ng/mL) and two quality control (QC) samples at 6 ng/mL and 15 ng/mL, which were run alongside all test samples. At least 10% of samples were injected in duplicate as an additional quality check. Data were collected using electrospray ionization (ESI) in negative mode using a Thermo Orbitrap Fusion mass spectrometer (Thermo Fisher Scientific, Waltham, MA). Chromatograms were generated using the monoisotopic mass of the [M-H]- ion for each analyte. Calibration curves were prepared as 1/x weighted quadratic fit using internal standard corrected peak areas. The method reporting limit (MRL) for each curve was set at the minimum value of the calibration curve where all calibration points had mean error < 20%. All resulting QC measurements were within the accepted 20% relative standard deviation range, except for PFDoA, which failed multiple QC criteria and was removed from reporting.

Chromatography was performed with a Vanquish Ultra-Performance Liquid Chromatography system (Thermo Fisher Scientific, Waltham, MA) on a Raptor C18 column (100 mm length x 2.1 mm diameter, 2.7 μm pore size). The flow rate was 350 μL/min with 25 μL injection volumes. Solvent A and Solvent B (95:5 acetonitrile:H_2_O, 2.5 mM ammonium acetate) made up the binary mobile phase gradient at various ratios depending on time point (Supplemental Table 2). Instrument settings and specifications are listed in Supplemental Table 3. In addition to targeted analysis, samples of all sea lions were analyzed in triplicate for non-targeted analysis (NTA) (Bangma et al., 2024). NTA features were prioritized based on PFAS signatures in mass spectrometry, including negative mass defect, the presence of fluorinated fragments in MS/MS, fluorine containing neutral losses, molecular formula generation, or partial matches to library spectra. A minimum abundance threshold was determined using the average abundance of a one ppb standard calibrant mix of all standards used in the targeted analysis. Our manual review of prioritized features identified no evidence of additional PFAS above the minimum abundance threshold. Based on our methods we would not expect detection of certain PFAS precursors incompatible with electrospray ionization (e.g. fluorotelomer alcohols), but have previously demonstrated detection of other LC-MS amenable species (e.g. zwitterions, novel anionic PFAS) (Bangma et al., 2024; Boettger et al., 2025).

### 2.3. Statistical analysis

Differences in serum PFAS concentrations were assessed between healthy GSL and CSL pups. Sea lions were then divided into four groups: 1) GSL juveniles, 2) GSL pups, 3) healthy CSL pups, and 4) malnourished CSL pups. Differences were assessed between these groups and between rookeries/islands in the Galapagos archipelago. Boxplots were generated to compare PFAS levels between groups and islands. Due to the differences in age between some groups, we also report age-adjusted means in Supplemental Table 5. Summary statistics (mean, standard deviation, median, minimum, maximum) are also reported for each group and each rookery. PFAS with at least 50% of samples at or above the MRL in each group were selected for comparative analysis (Table 2). Values below the MRL for these compounds were imputed by dividing the MRL by the square root of two (Hornung and Reed, 1990). A sensitivity analysis was performed to ensure stability of significance across different imputation methods. Values below the MRL were replaced with zero and with the MRL divided by two, both of which yielded slightly different point estimates but the same statistical significance. Summed PFAS concentrations were computed by summing the concentrations of all PFAS above the MRL (does not include imputed values). Serum PFAS concentrations were compared between healthy pups of each species using analysis of covariance (ANCOVA), accounting for the effect of age, sex, weight, and length, with an alpha level of 0.05 as the significance threshold. Serum PFAS concentrations were also compared between the four groups, between islands in the Galapagos archipelago (only islands with at least five samples), and between grouped inhabited and uninhabited islands in the archipelago in separate ANCOVA models with the same covariates and significance threshold as the previous sentence. In addition to the standard ANCOVA, a linear mixed effects model with island as a random effect was created to assess the impact of island on exposure between GSL. The assumptions of normality of residuals, homogeneity of variance, and homogeneity of regression slopes were checked and met. To control for the family-wise error rate, significant differences between groups, islands, and species were assessed using post hoc Tukey HSD tests for compounds that showed a significant effect of group or island in the ANCOVA model. Significant morphometric or signalment parameters in the ANCOVA were also further analyzed using Pearson’s correlation to assess the direction and strength of the relationships. All analysis was performed in R version 2024.04.1 + 748. R packages tidyverse (Wickham and e. a., 2019), dplyr (Wickham et al., 2023), and ggplot2 (Wickham, 2016) were utilized for all analyses.

## 3. Results

### 3.1. PFAS abundance

**Fig. 2.**
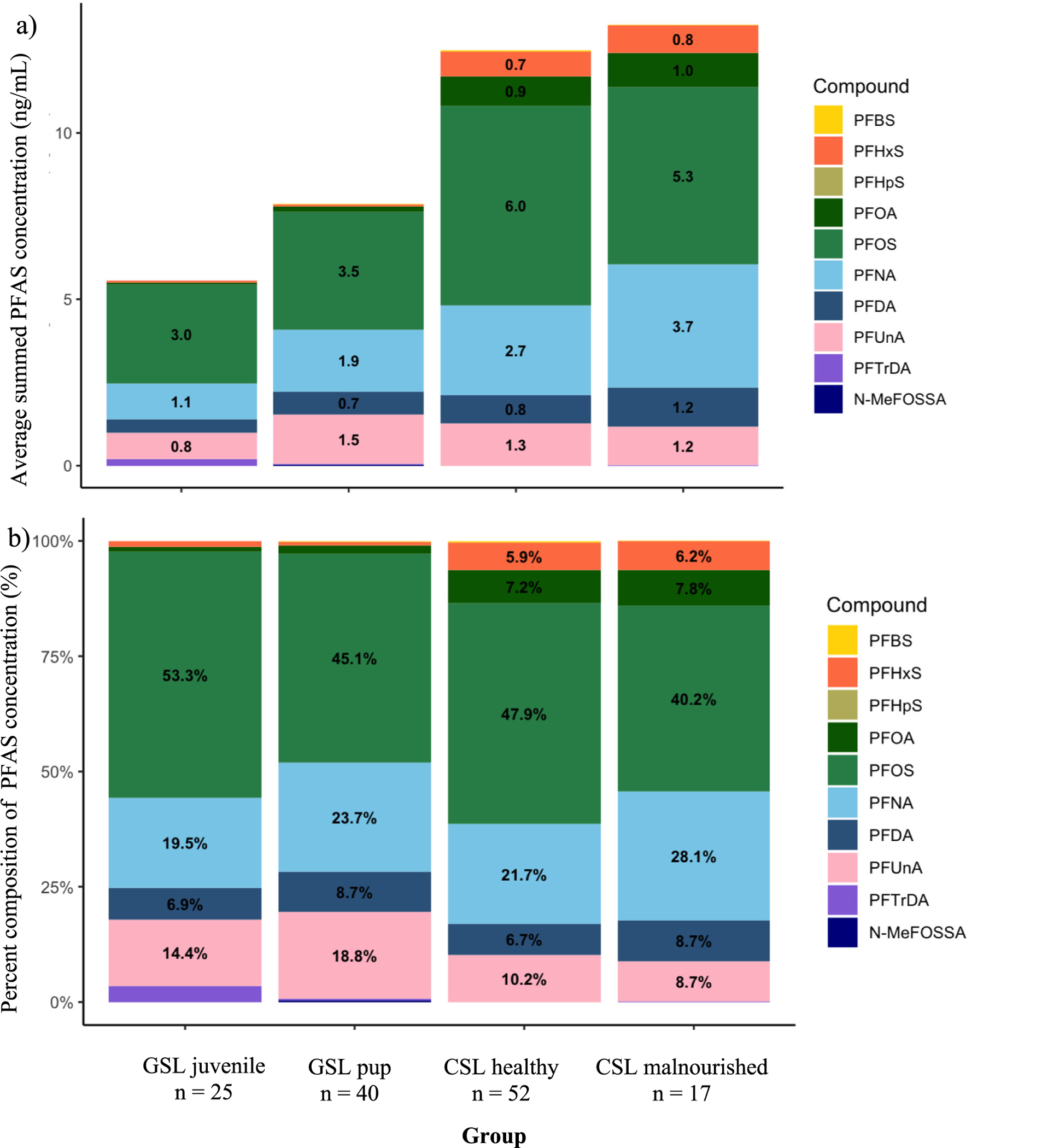
Average composition of summed serum PFAS concentrations in Galapagos sea lions (pups and juveniles) [GSL] (*Zalophus wollebaeki*) and California sea lions (healthy and malnourished pups) [CSL] (*Zalophus californianus*). Values below the method reporting limit (MRL) were treated as 0 when calculating the averages on this figure. PFAS with any observations above the MRL in any sea lion are included in this figure, as opposed to statistical analysis where only PFAS that were above the MRL in at least 50% of individuals were included. (a) Mean total PFAS concentrations by group. (b) Percentage of total summed PFAS concentrations by group.

PFOS was the most abundant compound in both species, with serum concentrations above the MRL for all specimens tested (Table 2). The proportion of specimens above the MRL for detected PFAS for each sampling location in the Galapagos archipelago is shown in Supplemental Table 4. In general, PFOS also had the highest mean concentrations in each group, followed by PFNA, PFUnA, PFDA, PFOA, and PFHxS (Table 3, Fig. 2a). The three exceptions to this trend are: 1) healthy CSL pups had a slightly higher mean serum concentration of PFOA compared to PFDA, 2) malnourished CSL pups had equal levels of PFUnA and PFDA, and 3) GSL pups and juveniles had mean concentrations of PFOA and PFHxS below the MRL. As a proportion of the total summed PFAS composition, CSL had higher PFOA and PFHxS than GSL, and GSL had higher PFUnA than CSL (Fig. 2b). The compounds PFNA, PFHxS, PFDA, and PFOA were above the MRL in over 95% of the CSL samples (Table 2). The proportion of each of these compounds above the MRL was lower in GSL. PFUnA was above the MRL in over 80% of CSL and GSL samples (Table 2). PFBA, PFPeA, 4:2FTS, PFHxA, PFPeS, PFHpA, 6:2FTS, PFOSA, PFNS, 8:2FTS, PFDS, PFTeDA, and N-EtFOSAA were not above the MRL in any group. PFBS, PFHpS, N-MeFOSAA, and PFTrDA were above the MRL in too few samples (<50%) to include in our comparative analysis. Of these, the only compounds that had over 10% of samples above the MRL were PFTrDA in GSL juveniles and PFBS in healthy CSL pups (Table 2). PFOA and PFHxS were also above the MRL in too few GSL samples (<50%), so they were not included in comparisons with CSL or in an assessment of PFAS across the Galapagos archipelago. Finally, N-MeFOSAA and PFHpS were only detected above the MRL in samples from GSL (Table 2). The average PFAS concentrations for each island are listed in Supplemental Table 6.

### 3.2. Inter- and intraspecific differences in exposure to PFAS

There were significant differences in PFAS exposure when comparing healthy pups of both species. PFOS (F = 24.10, *p* < 0.01), PFNA (F = 6.59, *p* = 0.01), and summed PFAS (F = 15.70, p < 0.01) varied significantly between species in an ANCOVA model. In the post hoc Tukey HSD test, CSL had higher concentrations of each of these compounds than GSL (Table 4).

**Fig. 3.**
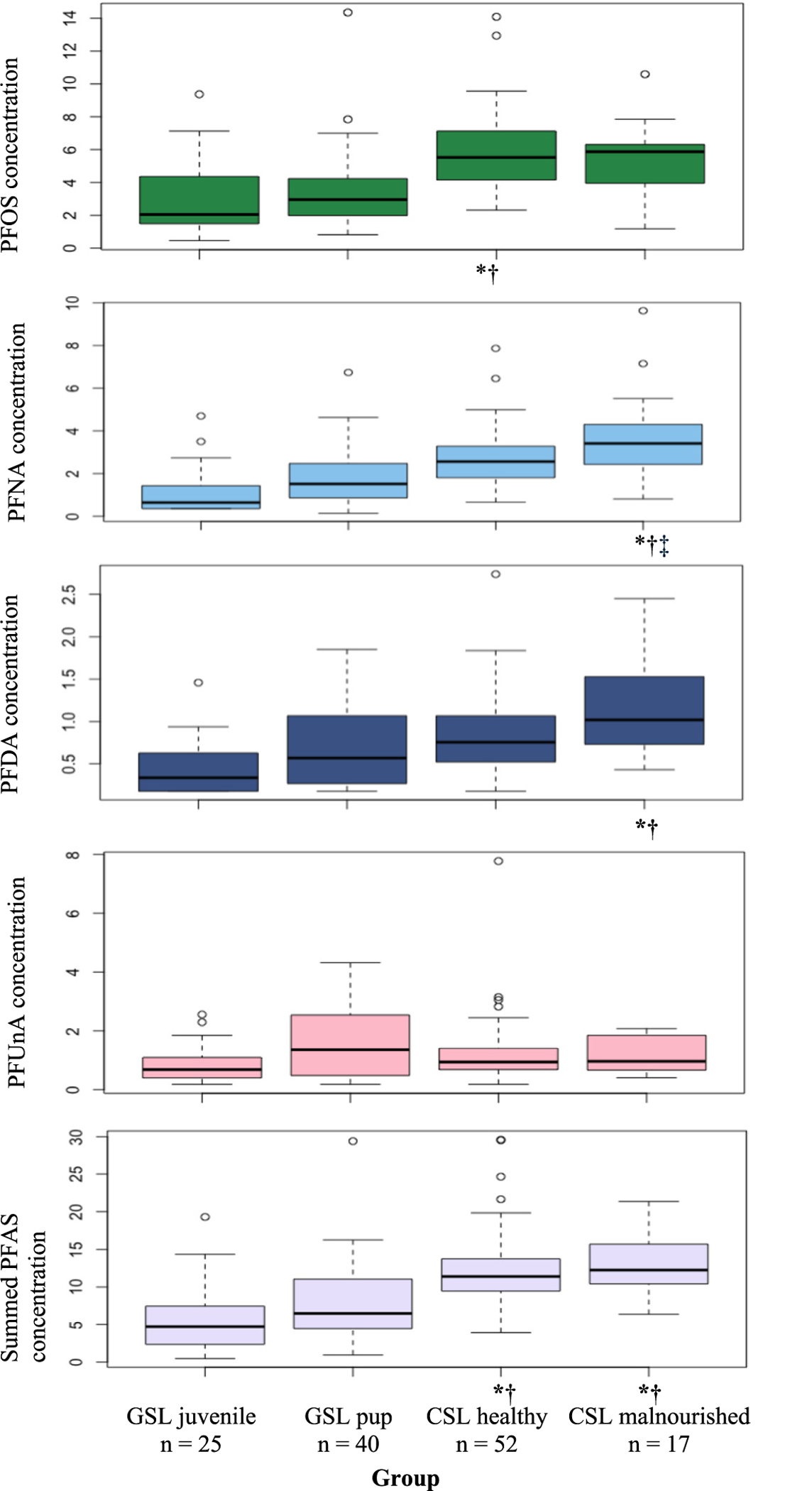
Box plots of serum PFAS concentrations of Galapagos sea lions (pups and juveniles) [GSL] (*Zalophus wollebaeki*) and California sea lions (healthy and malnourished pups) [CSL] (*Zalophus californianus*). Concentrations listed in ng/mL. Colors reflect those in Fig. 2.*Significantly greater than GSL juvenile. †Significantly greater than GSL pup. ‡Significantly greater than CSL healthy.

When analyzing all groups, PFOS (F = 9.48, p < 0.01), PFNA (F = 8.16, p <0.01), PFDA (F = 4.33, *P* <0.01), and summed PFAS (F = 9.38, p < 0.01) varied significantly by group in an ANCOVA model (Table 5 and Fig. 3). In follow-up Tukey HSD testing, summed serum PFAS concentrations were significantly higher in healthy and malnourished CSL pups than in GSL pups and juveniles (Table 5, Fig. 3). PFOS was significantly higher in healthy CSL pups than in GSL pups and juveniles. PFDA and PFNA were significantly higher in malnourished CSL than in GSL pups and juveniles, and PFNA was also significantly higher in malnourished CSL than in healthy CSL (Table 5). While this was the only significant intraspecific group difference observed, all compounds included in our comparative analysis were higher on average in GSL pups than in GSL juveniles, and most compounds tended to be higher in malnourished CSL pups than in healthy CSL pups (Table 3, Fig. 3).

**Fig. 4.**
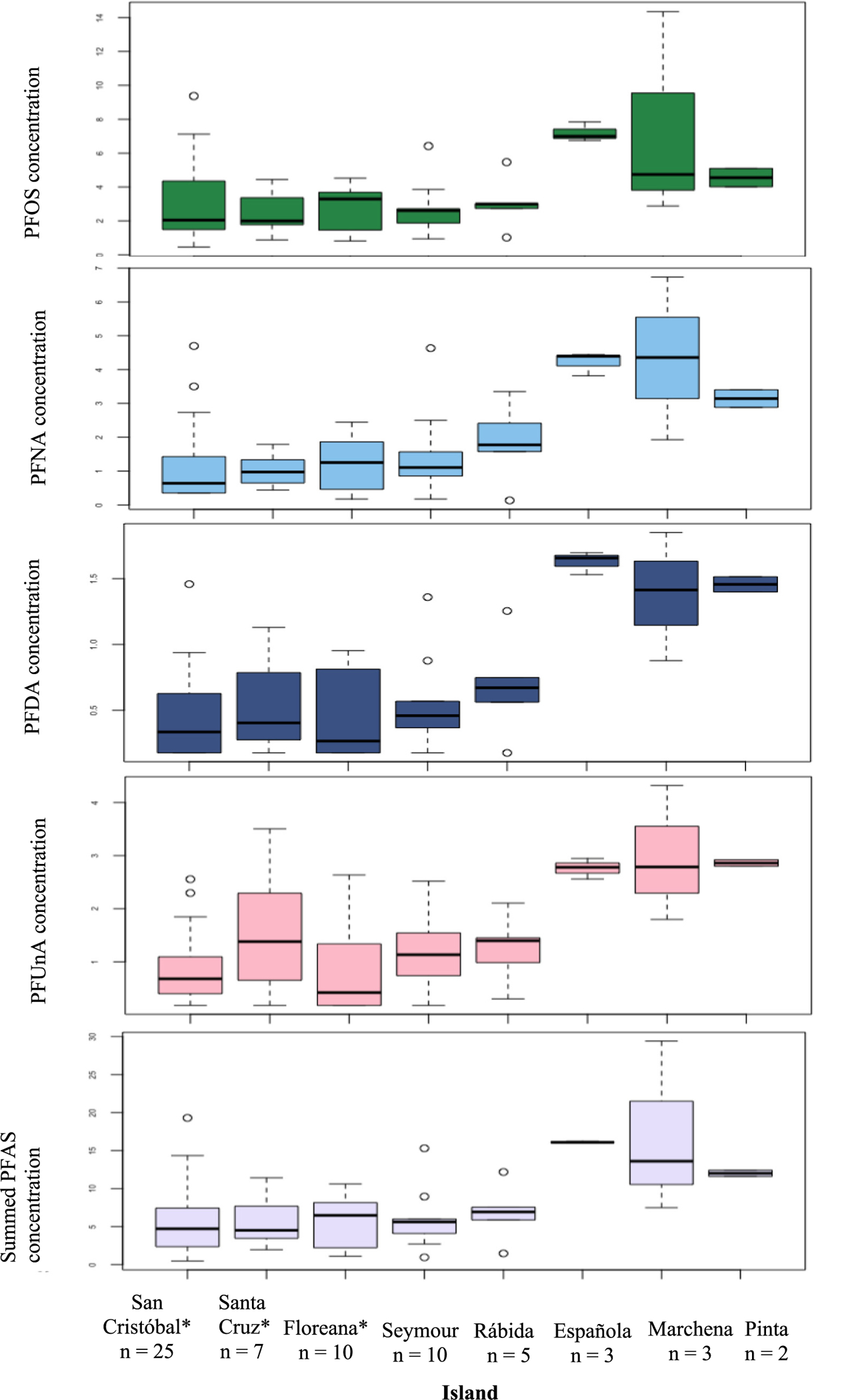
Box plots of serum PFAS concentrations in Galapagos sea lions (*Zalophus wollebaeki*) from various islands in the Galapagos archipelago. Concentrations listed in ng/mL. Colors reflect those in Fig. 2.*Island is inhabited by humans.

Differences in PFAS exposure between islands within the Galapagos archipelago were observed. Average concentrations from sea lions on the uninhabited islands Marchena, Española, and Pinta tended to have higher concentrations of PFAS than other islands, but there were too few samples from these islands to include in an ANCOVA assessing differences between islands. With those islands removed, there was no statistical evidence for differences in PFAS levels between individual islands (Fig. 4). In the follow-up linear mixed-effects model, the only compound for which island explained more than 1% of variation in exposure was PFDA, for which 10.4% of variation was explained by island. This is consistent with the ANCOVA results. In both models of island exposure variation in GSL, length showed high collinearity with other predictors and was excluded to improve model stability.

**Fig. 5.**
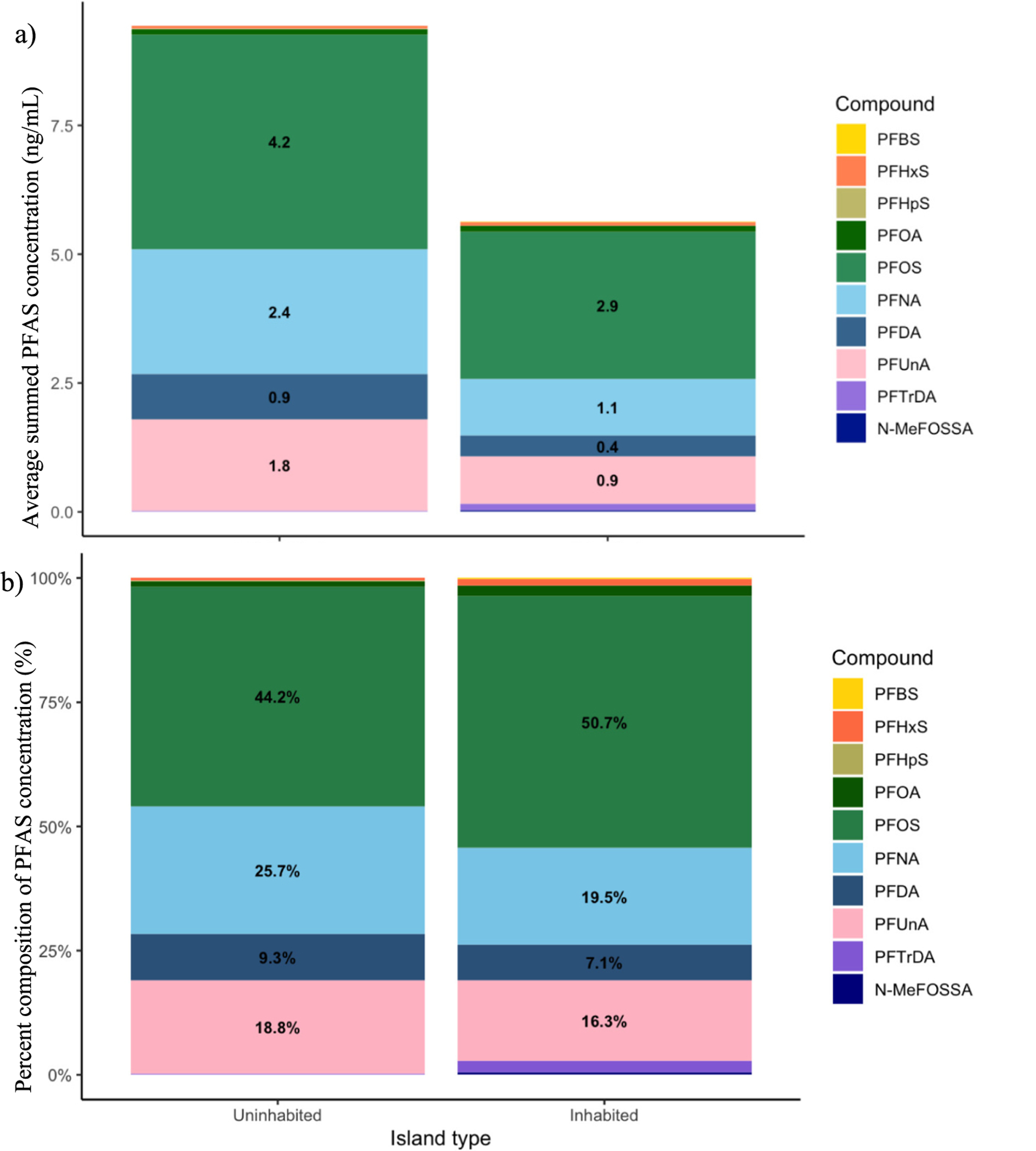
Average composition of summed serum PFAS in Galapagos sea lions [GSL] (*Zalophus wollebaeki*) on inhabited and uninhabited islands in the Galapagos archipelago. Values below the method reporting limit (MRL) were treated as 0 when calculating the averages on this figure. PFAS with any observations above the MRL in any sea lion are included in this figure, as opposed to statistical analysis where only PFAS that were above the MRL in at least 50% of individuals were included. (a) Mean total PFAS concentrations by group. (b) Percentage of total summed PFAS concentrations by group.

**Fig. 6.**
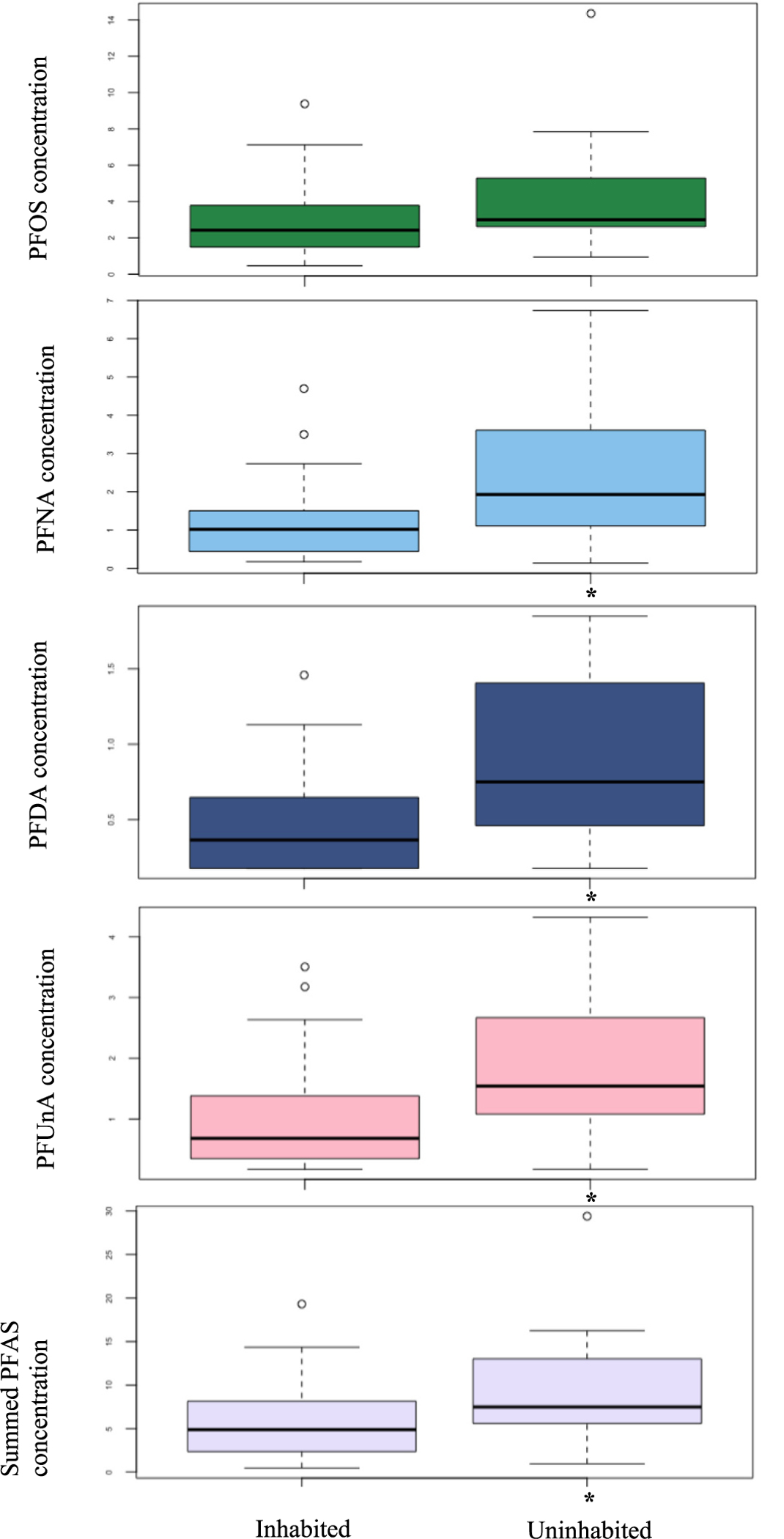
Box plots of serum PFAS concentrations in Galapagos sea lions (*Zalophus wollebaeki*) from inhabited and uninhabited islands in the Galapagos archipelago. Concentrations listed in ng/mL. Colors reflect those in Fig. 2. *PFAS concentration is significantly higher on uninhabited islands.

While individual islands had no significant impact on exposure, when grouping inhabited and uninhabited islands together, PFNA (F = 9.80, *p* < 0.01), PFDA (F = 9.19, p < 0.01), PFUnA (F = 5.91, *p* = 0.02), and summed PFAS (F = 5.14, *p* = 0.03) differed significantly between uninhabited and inhabited islands. The composition of PFAS exposure between inhabited and uninhabited islands is shown in Fig. 5. In follow-up Tukey HSD testing, uninhabited islands had significantly higher concentrations than inhabited islands for these compounds (Table 6, Fig. 6).

### 3.3. Associations between serum PFAS concentrations with signalment and morphometric data

In the ANCOVA model including healthy pups of both species, PFOS varied significantly by weight (F = 4.52, *p* = 0.04). In a follow-up analysis of correlation, PFOS was moderately positively correlated with weight when including both species (*r* = 0.44, p < 0.01). When assessing the correlation between weight and serum PFOS levels in the healthy pups of each species separately, only CSL had a significant positive correlation (*r* = 0.33, *p* = 0.02). There were no other significant differences in PFAS levels related to sex, weight, length, or age.

In the ANCOVA model including all groups, PFUnA varied significantly by weight (F = 6.14, *p* = 0.01). This was not significant in follow-up correlation analysis, suggesting there is no direct linear relationship between PFUnA and weight in the absence of the other covariates. An ANCOVA of PFOA and PFHxS was also conducted to compare healthy CSL vs malnourished CSL, since these groups each had sufficient sample numbers that were > 50% over the MRL. The two groups were not significantly different from each other for either compound. However, PFHxS varied significantly by length (F = 4.24, p = 0.04) and estimated age (F = 5.92, p = 0.02). Neither association was significant in the follow-up correlation analysis.

## 4. Discussion

Serum PFAS concentrations were generally higher in CSL than GSL, which is likely due to increased PFAS contamination in the ocean waters adjacent to California because of industrial processes. There are numerous military sites, airports, wastewater treatment plants, and industrial facilities off this coastline, which have contributed historically and currently to PFAS in the marine environment (Salvatore et al., 2022). Both otariid species showed similar relative abundances of PFAS, with PFOS being the most abundant compound. A few differences observed are that CSL tended to have a higher proportion of PFOA and PFHxS than GSL, and GSL tended to have a higher proportion of PFUnA than CSL. The higher PFUnA may be related to an increased regional production/use of this compound in South America, as marine PFAS concentrations generally reflect inputs from the nearest landmass (Khan et al., 2023). Previous studies of PFAS exposure in other marine mammals also reported PFOS to be the most abundant compound and reported similar relative abundances of the other compounds (Lopez-Berenguer et al., 2020; Shaw et al., 2009; Taylor et al., 2021; Boisvert et al., 2019). A prior 2001 study of PFOS in the livers of six adult CSL found higher concentrations than the present study (mean = 26.6 ng/g wet weight), however this is not directly comparable given that the matrix is different (Kannan et al., 2001). To our knowledge, there are only two prior studies of serum PFAS in pinnipeds: one with freshwater Baikal seals (*Pusa sibirica*), and one with harbor seals (*Phoca vitulina*) (Sait et al., 2023; Ishibashi et al., 2008; Sedlak et al., 2017; Sedlak and Greig, 2012). Mean serum PFOS, PFNA, and PFDA concentrations were slightly higher in healthy CSL pups in the current study than the 44 *P. sibirica* adults sampled in 2005, though their standard deviations overlapped (Ishibashi et al., 2008). Healthy CSL pups also had between 2–3× higher mean serum concentrations of PFOA than was reported for *P. sibirica* (Ishibashi et al., 2008). PFUnA was the only compound slightly higher in *P. sibirica* than in CSL, and again, the standard deviations overlapped (Ishibashi et al., 2008).

These differences in PFAS exposure could be due to a variety of factors, including age, time of sampling, differential exposure, and differential metabolism. While no age differences were reported in *P. sibirica*, other studies have reported an inverse relationship between PFAS exposure and age in marine mammals (Houde et al., 2005; Tao et al., 2006; Gui et al., 2019; Kannan et al., 2001; Shaw et al., 2009; Ishibashi et al., 2008). The individuals of *P. sibirica* were 11 years old on average, which may factor into their generally lower PFAS levels than the CSLs from this study. However, PFAS levels were higher (at least in the US human population) 15 years ago when the study of *P. sibirica* was performed (Ishibashi et al., 2008; Sonnenberg et al., 1999–2018). Because PFAS levels in marine environments generally reflect levels of the nearest landmass over time, levels in CSL may have been even higher at the time of the *P. sibirica* sampling (Khan et al., 2023). More work is needed to assess archived serum samples housed at marine mammal rehabilitation and research facilities to assess if burdens are decreasing in CSL over time off the Southern California coast, which may support the value and efficacy of recent policies surrounding PFAS. A separate assessment of PFAS plasma concentrations in *P. sibirica* (16 pups and two adult females) samples in 2011 found PFUnA (11.2 ng/g) to be more abundant than PFOS (8.67 ng/g) (Sait et al., 2023). The plasma concentrations of both compounds, as well as PFNA (4.65 ng/g) and PFDA (5.13 ng/g), were higher in the plasma of the 18 investigated *P. sibirica (*Sait et al., 2023) than the serum concentrations in each group reported in the current study. PFOA was the only compound higher in the CSL in this study than in the *P. sibirica* sampled in 2011 (Sait et al., 2023).

On the other hand, pups of *P. vitulina* in California’s San Francisco Bay showed much higher serum concentrations of PFOS, PFHxS, and PFOA than those detected in the current study (Sedlak et al., 2017; Sedlak and Greig, 2012). The level of PFOS detected in *P. vitulina* was over 30× greater than CSL and 55× greater than the GSL in the present study. This may be attributed to the fact that *P. vitulina* populations could be exposed to greater contamination from industrial facilities and former military bases (Sedlak et al., 2017; Sedlak and Greig, 2012). The differences may again be related to the fact that the sampling of *P. vitulina* in the San Francisco Bay occurred more than 10 years ago, when PFAS concentrations may have been higher in general (Sonnenberg et al., 1999–2018). In the current study, GSL had lower mean levels of all PFAS included in this analysis than *P. vitulina* and *P. sibirica (*Sait et al., 2023; Ishibashi et al., 2008; Sedlak et al., 2017; Sedlak and Greig, 2012). GSL juveniles in the current study also had average serum PFNA, PFDA, PFUnA, PFOS, and summed PFAS overlap (within a standard deviation) with plasma concentrations reported in the recent study of 14 GSL juveniles from the El Malecón rookery (the same rookery that the GSL juveniles in this study were collected) (Mehdi et al., 2025).

While the use and production of PFOA and PFOS are being phased out in the US, they can last for years in the body and other PFAS can break down into these two compounds (Zahm et al., 2024). Thus, this report is unable to distinguish whether exposures observed in CSL and GSL reflect past PFAS use, ongoing contamination, or a combination of both. Analyzing archived serum samples from these two species over the past few decades would be a necessary step in establishing temporal trends and assessing the effectiveness of current PFAS mitigation efforts.

Prior assessments of PFAS in marine mammals have reported a negative association between various compounds and age, weight, and length (Houde et al., 2005; Tao et al., 2006; Gui et al., 2019; Kannan et al., 2001; Shaw et al., 2009; Ishibashi et al., 2008). This negative association is likely due to in-utero and lactational exposure leading to higher concentrations in younger individuals. For example, in-utero and lactational PFOA exposure has been documented in both humans and animals and has been associated with low birth weights and the development of chronic diseases in adulthood (Blake and Fenton, 2020). In-utero exposure to various PFAS has also been documented in neonates of the Australian sea lion (*Neophoca cinerea),* Australian fur seal (*Arctocephalus pusillus),* and Baikal seal (Taylor et al., 2021; Ishibashi et al., 2008). Of interest, while they are lactating, adult female pinnipeds undertake short migrations to feed, suggesting that PFAS contaminants located close to the rookery would represent the majority of PFAS in the serum of their pups (Trillmich, 1979; Melin et al., 2000; Peterson and Bartholomew, 1967). Another difference worth noting between GSL and CSL is their stark difference in their weaning times. On average, CSL weans in under a year and GSL may take up to three years to fully wean, with CSL incorporating some fish into their diet as early as three months and GSL not incorporating fish until at least a year (Jeglinski et al., 2012; Trillmich and Wolf, 2008; Rosas-Hernández et al., 2024). Since PFAS can be obtained through lactation and diet, these differences between GSL and CSL could also play a role in their differential exposure profiles. Additionally, PFOS was positively correlated with weight in healthy pups in the current study. This may be related to the limited age range. Further work including more age classes would be needed to assess trends between PFAS exposure and morphometric parameters in these species. Additionally, no sex differences for any of the PFAS were observed in this study, as has been reported in other marine mammal species (Lopez-Berenguer et al., 2020; Sait et al., 2023; Boisvert et al., 2019).

The analysis of serum PFAS in GSL revealed differences in PFAS concentrations between islands, where animals sampled at islands uninhabited by humans had significantly higher levels of PFNA, PFDA, PFUnA, and summed PFAS than islands inhabited by humans. This is particularly interesting since GSL exhibit foraging site fidelity and adults may not commonly move between regions in the archipelago (Drago et al., 2016). There are many factors that could be related to differential exposures between islands. One of those factors are the wind and ocean currents coming from the mainland. Currents have been shown to play a major role in PFAS transportation in a marine environment (Ma et al., 2025). Española island in the southeast and Marchena and Pinta islands in the north of the archipelago face the brunt of the Peru and Panama oceanic currents, respectively (Forryan et al., 2021; Palacios et al., 2006), and are those with the highest PFAS concentrations. There were only two or three samples from each of these islands, and more samples would be required to fully understand the differences on these islands. Prior work investigating plastic pollution in the archipelago demonstrated that, as expected, windward sides of islands have more pollution than leeward sides of islands, supporting the hypothesis that various contaminants may originate from the mainland (Muñoz-Pérez et al., 2023). Other studies have reported that Marine Protected Areas have the same levels of plastic pollution as non-protected areas (Botterell et al., 2024) and that pollutants are present across the Galápagos archipelago, including islands not inhabited by humans, in a variety of species (Savage et al., 2024). Prior work has also demonstrated that atmospheric PFAS may be responsible for PFAS contamination in remote locations (Martin et al., 2006).

Another factor with the potential to contribute to higher PFAS observed on uninhabited islands are dietary differences between islands. Prior work has demonstrated that GSL on different islands have differing diets and foraging sites around the archipelago, though their trophic level remains the same (Drago et al., 2016; Páez-Rosas and Aurioles-Gamboa, 2014; Urquía and Páez-Rosas, 2019). If there are differences in PFAS burden in the fish that sea lions at different sites are consuming, this would likely be reflected in the sea lions. One would expect to see more variation in the longer-chain PFAS (like the PFNA, PFDA, and PFUnA that did differ significantly between inhabited and uninhabited islands) to show the most diet-related variation as these are the compounds that exhibit the most biomagnification and tend to have higher concentrations in marine apex predators (Khan et al., 2023).

The last factor to be discussed here, though there are likely many more variables at play, is the population structure of breeding females at different islands in the archipelago. While the data do not exist on the ages of females in different rookeries, reports have shown that the population structure (including pup abundance) does vary between rookeries (Riofrío-Lazo et al., 2017). The ages of the mothers at these rookeries could be relevant because parity may influence the maternal offloading of PFAS as it does in humans (Hall et al., 2022).

Despite being located within a human settlement (and thus, the pollution associated with humans), the lowest concentrations of PFAS were observed in the juvenile sea lions sampled on San Cristobal Island. The sea lions from this island were sampled from the El Malecón rookery, which is home to the largest sea lion population of the archipelago (Páez-Rosas et al., 2021; Riofrío-Lazo et al., 2017). This may also be related to these juveniles being older than the pups sampled on other islands. However, further work is needed to investigate factors leading to differences between islands.

Serum PFNA and PFDA concentrations tended to be higher in malnourished CSL pups than in healthy CSL pups, with PFNA being significantly higher. While there havenť been any studies supporting the idea that PFAS exposure may contribute to the development of malnutrition (to our knowledge), there is prior literature supporting the hypothesis that altered physiologic states can alter PFAS exposure profiles in various wildlife species (Bangma et al., 2022). PFNA and PFDA tend to be higher in the liver compared to other PFAS in pinnipeds. For example, in one study on Baikal seals, the liver:serum ratios for levels of PFNA, PFDA, and PFOS were 14, 15, and 2.4, respectively (Ishibashi et al., 2008). This is likely due to PFNA and PFDA being longer-chain compounds than PFOS. These PFAS are also proteinophilic, suggesting that the tissue metabolism in malnourished pups may mobilize PFAS into the blood (Ng and Hungerbühler, 2013). Many PFAS, including PFNA, also bind to proteins such as albumin and fatty acid binding proteins in the serum and liver, respectively. Disease-associated and natural changes in physiology that alter the expression of these proteins have been associated with changes in several PFAS concentrations in wildlife (Bangma et al., 2022). Therefore, it is likely that changes in protein expression as well as lipid catabolism in malnourished sea lions are impacting the PFNA levels in the serum. It is important to note that the malnourished pups (aged ~11 mos.) were older than the healthy pups (aged ~6mos.) on average. Since PFAS typically decline with age in young mammals, their PFAS levels could have been lower on average before becoming malnourished (Houde et al., 2005; Tao et al., 2006; Gui et al., 2019; Kannan et al., 2001; Shaw et al., 2009; Ishibashi et al., 2008). It is also possible that this cohort did simply have higher exposures than the healthy pups, though this is unlikely considering that they are assumed to have originated from the same place (the Channel Islands of California). The difference in age between the healthy and malnourished pups is a major confounding variable, and comparing pups closer in age would be necessary to further investigate the potential impacts of malnutrition on sea lion pups.

Prior work has demonstrated impaired immunity and increased risk of disease associated with PFAS exposure, including in marine mammals. For example, an in-vitro analysis of bottlenose dolphins, *Tursiops truncatus*, demonstrated that PFOS exposure was associated with increased CD4+ and CD8+ T cell proliferation and induced proinflammatory cytokine production and proliferation at environmentally relevant levels (Soloff et al., 2017). Unfortunately, similar studies do not yet exist for pinnipeds to our knowledge. Both PFOA and PFOS have strong mechanistic evidence of being carcinogenic, with PFOA being immunosuppressive and inducing epigenetic changes (Zahm et al., 2024). While there is no established toxicity threshold for which levels of PFAS cause adverse effects in marine mammals, the National Academies of Sciences and Medicine’s Guidance on PFAS Exposure, Testing, and Clinical Follow-Up concluded that summed serum PFAS levels between two and 20 ng/mL have the potential for adverse effects in humans, while exposures over 20 ng/mL put one at increased risk for adverse effects (Academies, 2022). Of note, the average summed PFAS concentrations for each pinniped group in this study fell in the 2–20 ng/mL window, and the maximum summed PFAS concentration for each species was over 20 ng/mL (29.41 ng/mL for GSL and 29.60 ng/mL for CSL). A human reference for toxicity threshold is not ideal for evaluating potential risks to pinnipeds, as our physiology and life histories are very different, and more work is warranted to understand what exposure levels put marine mammals at risk of harmful effects.

Some of the potential harmful effects of PFAS exposure that most warrant further investigation in sea lions are its potential immunosuppressive and carcinogenic effects in these species. As mentioned before, CSL have the highest incidence of cancer among marine mammals (Gulland et al., 1996; Deming et al., 2018). In a study of 95 CSL with and 163 without UG carcinoma, OtHV1 was detected in 100% of sea lions with this cancer and in 36% of sea lions without it (Deming et al., 2021). The viral load was also lower in the CSL without UG carcinoma. This suggests that the state of the immune system may play an integral role in whether OtHV1 infection may lead to UG carcinoma. Another study showed that immunosuppressive persistent organic pollutants were also associated with UG carcinoma incidence in CSL (Gulland et al., n.d.). PFAS may be another class of chemicals that could potentially put CSL at risk for UG carcinoma by immune suppression. As mentioned before, a variety of PFAS (including PFOA and PFOS) have been associated with immune disruption in marine mammals and people (Kannan et al., 2006; Zahm et al., 2024; Soloff et al., 2017). More work is needed to understand the role that PFAS exposure might play in cancer development in marine mammals, particularly when those cancers have been linked to viruses.

The immunosuppressive nature of PFAS also warrants concern for the endangered GSL. GSL have been exposed to a number of introduced disease-causing pathogens including canine distemper virus, *Leptospira,* and *Diofilaria immitis (*Gregory et al., 2023; Denkinger et al., 2017). Stress-inducing events like El Niño and exposure to immunosuppressive chemicals can impair the body’s ability to respond to pathogens and thus can contribute to disease outbreaks in marine mammals (Desforges et al., 2016; Páez-Rosas et al., 2021; Ross, 2002). Disease outbreaks can prove detrimental in endangered species, including GSL. From 2009 to 2012 there was an unusual pup mortality event during a non-El Nino year where pup mortality rates reached 67% in some colonies with close contact to human settlement (Denkinger et al., 2017). While the cause of this unusual mortality event could not be confirmed, there was evidence for infectious disease. Leptospira was identified by PCR and sequencing in five out of seven tissue samples tested, and canine distemper virus was identified by PCR and sequencing in six of 48 tissue samples (Denkinger et al., 2017). Unusual mortality events such as this one in combination with El Nino-associated mortality events (which can have pup mortality rates of up to 100%) pose a substantial threat to the endangered GSL whose population has already declined by 50% in the last 40 years. Thus, any immune-disrupting contaminants that may increase the risk of disease outbreaks may pose a threat to this species. It is also possible that the malnutrition associated with El Nino events in GSL may mobilize PFAS into the bloodstream, with each stressor potentially worsening the impacts of the other (Bangma et al., 2022). Follow-up work with pinnipeds of similar age classes would be necessary to investigate this hypothesis.

## 5. Conclusions

PFAS levels were generally higher in serum samples of the CSL than in the GSL. While data do not yet exist to determine the levels of PFAS that lead to increased risk of adverse effects in pinniped species, serum levels associated with adverse effects in humans were observed in both species in this study. More work is necessary to determine whether PFAS exposure may contribute to disease development or adverse health outcomes in sea lions. If PFAS are associated with these adverse outcomes, CSL may serve as a warning bell for the endangered GSL. We suggest the following prioritized research actions: 1. Investigate the immunotoxic and carcinogenic effects of PFAS in pinnipeds; 2. Further investigate differences in PFAS exposure in pinnipeds in the Galapagos archipelago; 3. Investigate trends in PFAS exposure over the last few decades to assess the effectiveness of PFAS mitigation strategies. The presence of PFAS contaminants in one of the world’s most pristine environments, the Galapagos archipelago, draws into sharp focus the need for surveillance of environmental contaminants and research into their potential adverse health effects on its iconic species.

## Supplementary Material

Supplement1

## Figures and Tables

**Table 1:** Galapagos [GSL] (*Zalophus wollebaeki*) and California [CSL] (*Zalophus californianus*) sea lion serum samples analyzed for PFAS in this report. CSL healthy samples were collected in November 2022 (11 pups, approximately five months old), September 2023 (17 pups, approximately three months old), and March/April 2024 (24 pups, approximately nine mos old).

Population	n	Collection Year	Mean Age (months)*	Mean Length (cm)*	Percent Male	Location
GSL juvenile	25	2022	20 (6)	113.64 (16.26)	34.8%	San Cristobal Island
GSL pup	40	2024	6 (2)	94.87 (8.16)	62.5%	Across Galapagos archipelago
CSL healthy	52	2022–2024	6 (3)	104.45 (9.19)	58.8%	San Miguel Island
CSL malnourished	17	2022–2023	11 (5)	100.39 (9.48)	47.2%	Pacific Marine Mammal Center

* Parentheses represent standard deviation.

**Table 2:** Proportion of Galapagos sea lions (pups and juveniles) [GSL] (*Zalophus wollebaeki*) and California sea lions (healthy and malnourished pups) [CSL] (*Zalophus californianus*) above the method reporting limit (MRL) for each PFAS included in the targeted serum analysis. PFBA, PFPeA, 4:2FTS, PFHxA, PFPeS, PFHpA, 6:2FTS, PFOSA, PFNS, 8:2FTS, PFDS, PFTeDA, and N-EtFOSAA were not above the MRL in any sample and thus were left off this table. The MRL (ng/mL) of each compound is listed underneath the compound name.

Population	PFOS 0.5	PFNA 0.5	PFHxS 0.25	PFDA 0.25	PFUnA 0.25	PFOA 0.25	N-MeFOSAA 0.25	PFTrDA 0.5	PFHpS 0.25	PFBS 0.25
**GSL juvenile**	1.00	0.68	0.20	0.64	0.76	0.16	0.00	0.44	0.00	0.00
**GSL pup**	1.00	0.93	0.13	0.75	0.89	0.38	0.08	0.05	0.03	0.03
**CSL healthy**	1.00	1.00	0.98	0.98	0.81	0.98	0.00	0.00	0.00	0.12
**CSL malnourished**	1.00	1.00	1.00	1.00	1.00	0.94	0.00	0.06	0.00	0.06

**Table 3:** Summary of serum PFAS concentrations in Galapagos sea lions (pups and juveniles) [GSL] (*Zalophus wollebaeki*) and California sea lions (healthy and malnourished pups) [CSL] (*Zalophus californianus*). Compounds are listed in order of increasing carbon chain length. MRL = method reporting limit.

Compound	Group	n	Mean (ng/mL)	Standard Deviation	Median	Min	Max
PFHxS	GSL juvenile	25	< MRL		< MRL	< MRL	0.48
	GSL pup	40	< MRL		< MRL	< MRL	0.84
	CSL healthy	52	0.74	0.32	0.69	< MRL	2.32
	CSL malnourished	17	0.82	0.28	0.80	0.33	1.29
PFOA	GSL juvenile	25	< MRL		< MRL	< MRL	0.46
	GSL pup	40	< MRL		< MRL	< MRL	0.74
	CSL healthy	52	0.90	0.82	0.75	< MRL	5.36
	CSL malnourished	17	1.04	0.68	0.88	< MRL	2.58
PFOS	GSL juvenile	25	2.97	2.19	2.05	< MRL	9.37
	GSL pup	40	3.55	2.46	2.95	0.82	14.36
	CSL healthy	52	5.99	2.29	5.53	2.31	14.09
	CSL malnourished	17	5.33	2.35	5.87	1.18	10.59
PFNA	GSL juvenile	25	1.20	1.12	0.64	< MRL	4.70
	GSL pup	40	1.88	1.51	1.51	< MRL	6.74
	CSL healthy	52	2.71	1.37	2.56	0.66	7.87
	CSL malnourished	17	3.72	2.13	3.41	0.81	9.63
PFDA	GSL juvenile	25	0.45	0.33	0.34	< MRL	1.46
	GSL pup	40	0.73	0.51	0.57	< MRL	1.85
	CSL healthy	52	0.84	0.44	0.75	< MRL	2.74
	CSL malnourished	17	1.16	0.64	1.02	0.43	2.45
PFUnA	GSL juvenile	25	0.84	0.67	0.68	< MRL	2.56
	GSL pup	40	1.50	1.08	1.36	< MRL	4.32
	CSL healthy	52	1.27	1.14	0.94	< MRL	7.78
	CSL malnourished	17	1.16	0.58	0.96	0.40	2.08
Summed	GSL juvenile	25	5.57	4.65	4.71	0.46	19.31
	GSL pup	40	7.86	5.60	6.45	0.94	29.41
	CSL healthy	52	12.49	5.31	11.39	3.91	29.60
	CSL malnourished	17	13.24	4.32	12.26	6.34	21.38

**Table 4:** Summary of significant post hoc Tukey HSD test results comparing serum PFAS concentrations in healthy Galapagos sea lion [GSL] (*Zalophus wollebaeki*) pups and healthy California sea lion [CSL] (*Zalophus californianus*) pups. The difference in means is how much greater the mean of CSL is than the mean of GSL. Compounds are listed in order of increasing carbon chain length.

Compound	Difference in Means (ng/mL)	95% CI Lower	95% CI Upper	*p*-value
PFOS	2.38	1.41	3.34	<0.01
PFNA	0.73	0.16	1.29	0.01
Summed	4.29	2.14	6.45	<0.01

**Table 5:** Summary of significant post hoc Tukey HSD test results comparing serum PFAS concentrations in Galapagos sea lions (pups and juveniles) [GSL] (*Zalophus wollebaeki*) and California sea lions (healthy and malnourished pups) [CSL] (*Zalophus californianus*). Compounds are listed in order of increasing carbon chain length.

Compound	Comparison	Difference in Means (ng/mL)	95% CI Lower	95% CI Upper	p-value
PFOS	CSL healthy - GSL juvenile	2.71	0.66	4.77	<0.01
	CSL healthy - GSL pup	2.38	1.07	3.68	<0.01
PFNA	CSL malnourished - GSL juvenile	2.28	0.80	3.77	<0.01
	CSL malnourished - GSL pup	1.84	0.73	2.95	<0.01
	CSL malnourished - CSL healthy	1.11	0.04	2.19	0.04
PFDA	CSL malnourished - GSL juvenile	0.60	0.11	1.09	<0.01
	CSL malnourished - GSL pup	0.43	0.07	0.80	0.01
Summed	CSL healthy - GSL juvenile	5.60	1.23	9.97	<0.01
	CSL malnourished - GSL juvenile	6.69	1.60	11.78	<0.01
	CSL healthy - GSL pup	4.29	1.52	7.07	<0.01
	CSL malnourished - GSL pup	5.38	1.58	9.19	<0.01

**Table 6:** Summary of significant post hoc Tukey HSD test results comparing serum PFAS concentrations in Galapagos sea lions (*Zalophus wollebaeki*) from inhabited and uninhabited islands in the Galapagos archipelago. The difference in means is how much greater the mean of all uninhabited islands is than the mean of all inhabited islands included in this study. Compounds are listed in order of increasing carbon chain length.

Compound	Difference in Means (ng/mL)	95% CI Lower	95% CI Upper	p-value
PFNA	1.18	0.42	1.94	<0.01
PFDA	0.38	0.13	0.64	<0.01
PFUnA	0.67	0.12	1.23	0.02
Summed	3.37	0.38	6.37	0.03

## Data Availability

Data will be made available on request.

## Appendix A. Supplementary data

Supplementary data to this article can be found online at https://doi.org/10.1016/j.marpolbul.2026.119666.

## Glossary

PFA: Per- and polyfluoroalkyl substances
[GSL] (Zalophus wollebaeki): Galapagos sea lion
[CSL] (Zalophus californianus): California sea lion
