# Supplementary material for: Differences in PFAS exposure between Pacific pinnipeds: The Galapagos (Zalophus wollebaeki) and California (Zalophus californianus) sea lions
Source: Mar Pollut Bull. Author manuscript; Available in PMC 2026 Jul 23. (PMC13392999; doi:10.1016/j.marpolbul.2026.119666)
Supplement: Supplement1 [file NIHMS2169090-supplement-Supplement1.docx]

**Supplemental Table 1.** Full PFAS names, chemical abstracts service numbers (CAS), abbreviations, and matched internal standard (IS) of PFAS included in the targeted analysis of Galapagos (*Zalophus wollebaeki*) and California (*Zalophus californianus*) sea lion serum. Compounds are listed in order of increasing carbon chain length.

| **Compound** | **CAS** | **Acronym** | **IS Acronym** | **Internal Standard** | |
| --- | --- | --- | --- | --- | --- |
| Perfluorobutanoic acid | 375-22-4 | PFBA | MPFBA | | Perfluoro-n-(^13^C_4_)butanoic acid |
| Perfluorobutane sulfonic acid | 375-73-5 | PFBS | M3PFBS | | Sodium perfluoro-1-(2,3,4-^13^C_3_)butanesulfonate |
| Perfluoropentanoic acid | 2706-90-3 | PFPeA | MSPFPeA | | Perfluoro-n-(^13^C_5_)pentanoic acid |
| Perfluoropentanesulfonic Acid | 2706-91-4 | PFPeS | M3PFBS | | Sodium perfluoro-1-(2,3,4-^13^C_3_)butanesulfonate |
| 4:2 Fluorotelomer sulfonic acid | 757124-72-4 | 4:2FTS | M2-4:2FTS | | Sodium 1H, 1H,2H,2H-perfluoro-1-(1,2-^13^C_2_) hexanesulfonate |
| Perfluorohexanoic acid | 307-24-4 | PFHxA | M5PFHxA | | Perfluoro-n-(1,2,3,4,6-^13^C_5_)hexanoic acid |
| Perfluorohexane sulfonate | 108427-53-8 | PFHxS | M3PFHxS | | Sodium perfluoro-1-(1,2,3-^13^C_3_)hexanesulfonate |
| Perfluoroheptanoic acid | 375-85-9 | PFHpA | M4PFHpA | | Perfluoro-n-(1,2,3,4-^13^C_4_)heptanoic acid |
| Perfluoroheptanesulfonic acid | 375-92-8 | PFHpS | M3PFHxS | | Sodium perfluoro-1-(1,2,3-^13^C_3_)hexanesulfonate |
| 6:2 Fluorotelomer sulfonic acid | 27619-97-2 | 6:2FTS | M2-6:2FTS | | Sodium 1H, 1H,2H,2H-perfluoro-1-(1,2-^13^C_2_) octanesulfonate |
| Perfluorooctanoic acid | 335-67-1 | PFOA | M8PFOA | | Perfluoro-n-(^13^C_8_)octanoic acid |
| Perfluorooctanesulfonic acid | 1763-23-1 | PFOS | M8PFOS | | Sodium perfluoro-1-(^13^C_8_)octanesulfonate |
| Perfluorooctanesulfonamide | 754-91-6 | PFOSA | M8FOSA | | Perfluoro-1-(^13^C_8_)octanesulfonamide |
| Perfluorononanesulfonic acid | 68259-12-1 | PFNS | M8PFOS | | Sodium perfluoro-1-(^13^C_8_)octanesulfonate |
| Perfluorononanoic acid | 375-95-1 | PFNA | M9PFNA | | Perfluoro-n-(^13^C_9_)nonanoic acid |
| 8:2 Fluorotelomer sulfonic acid | 39108-34-4 | 8:2FTS | M2-8:2FTS | | Sodium 1H, 1H,2H,2H-perfluoro-1-(1,2-^13^C_2_) decanesulfonate |
| Perfluorodecanoic acid | 335-76-2 | PFDA | M6PFDA | | Perfluoro-n-(1,2,3,4,5,6-^13^C_6_)decanoic acid |
| Perfluorodecane sulfonic acid | 335-77-3 | PFDS | M8PFOS | | Sodium perfluoro-1-(^13^C_8_)octanesulfonate |
| N-methylperfluorooctane  sulfonamidoacetic acid | 2355-31-9 | N-MeFOSAA | d3-N-MeFOSAA | | N-Methyl-d_3_ -perfluoro-1-octanesulfonamidoacetic acid |
| Perfluoroundecanoic acid | 2058-94-8 | PFUnA | M8PFOS | | Sodium perfluoro-1-(^13^C_8_)octanesulfonate |
| N-Ethylperfluorooctane  sulfonamidoacetic acid | 2991-50-6 | N-EtFOSAA | d5-N-EtFOSAA | | N-Ethyl-d_5_-perfluoro-1-octanesulfonamidoacetic acid |
| Perfluorotridecanoic acid | 72629-94-8 | PFTrDA | M8PFOS | | Sodium perfluoro-1-(^13^C_8_)octanesulfonate |
| Perfluorododecanoic acid | 307-55-1 | PFDoA | MPFDOA | | Perfluoro-n-(1,2-^13^C_2_)dodecanoic acid |
| Perfluorotetradecanoic acid | 376-06-7 | PFTeDA | M2PFTeDA | | Perfluoro-n-(1,2-^13^C_2_) tetradecanoic acid |

**Supplemental Table 2.** Mobile phase compositions for liquid chromatography.

| **Time (mins)** | **%B** |
| --- | --- |
| -1.50 | 25 |
| -0.01 | 25 |
| -0.01 | 25 |
| 0.00 | 25 |
| 1.00 | 25 |
| 2.00 | 25 |
| 5.00 | 80 |
| 8.50 | 100 |
| 11.50 | 100 |

**Supplemental Table 3.** Thermo Orbitrap Fusion mass spectrometer instrument settings.

| Application Mode | Small Molecule |
| --- | --- |
| Resolution | 50,000 |
| Scan range | 120-1200 |
| RF lens % | 60 |
| Ion source type | H-ESI |
| Polarity | Negative |
| Voltage | 2800 |
| Gas Mode | Static |
| Sheath Gas (Arb) | 40 |
| Aux Gas (Arb) | 5 |
| Sweep Gas (Arb) | 2.2 |
| Ion transfer Tube Temp (C) | 300 |
| Vaporizer Temp (C) | 200 |

**Supplemental Table 4.** Proportion of Galapagos sea lion (*Zalophus wollebaeki*) serum samples above the MRL for each PFAS included in the targeted analysis. PFBA, PFPeA, 4:2FTS, PFHxA, PFPeS, PFHpA, 6:2FTS, PFOSA, PFNS, 8:2FTS, PFDS, PFTeDA, and N-EtFOSAA were not above the MRL in any sample included in the analysis.

| **Island** | **PFOS** | **PFNA** | **PFHxS** | **PFDA** | **PFUnA** | **PFOA** | **N-MeFOSAA** | **PFTrDA** | **PFHpS** | **PFBS** |
| --- | --- | --- | --- | --- | --- | --- | --- | --- | --- | --- |
| **San Cristobal*** | 1.00 | 0.68 | 0.20 | 0.64 | 0.76 | 0.16 | 0.00 | 0.44 | 0.00 | 0.00 |
| **Santa Cruz*** | 1.00 | 1.00 | 0.00 | 0.71 | 0.86 | 0.29 | 0.00 | 0.14 | 0.00 | 0.00 |
| **Floreana*** | 1.00 | 0.80 | 0.30 | 0.50 | 0.70 | 0.70 | 0.30 | 0.00 | 0.00 | 0.10 |
| **Seymour** | 1.00 | 0.90 | 0.10 | 0.80 | 0.90 | 0.10 | 0.00 | 0.00 | 0.00 | 0.00 |
| **Rábida** | 1.00 | 1.00 | 0.00 | 0.80 | 1.00 | 0.00 | 0.00 | 0.00 | 0.00 | 0.00 |
| **Española** | 1.00 | 1.00 | 0.00 | 1.00 | 1.00 | 1.00 | 0.00 | 0.00 | 0.00 | 0.00 |
| **Marchena** | 1.00 | 1.00 | 0.33 | 1.00 | 1.00 | 0.67 | 0.00 | 0.33 | 0.33 | 0.00 |
| **Pinta** | 1.00 | 1.00 | 0.00 | 1.00 | 1.00 | 0.00 | 0.00 | 0.00 | 0.00 | 0.00 |

**Supplemental Table 5.** Age-adjusted mean serum PFAS concentrations in Galapagos sea lions (pups and juveniles) [GSL] (*Zalophus wollebaeki*) and California sea lions (healthy and malnourished pups) [CSL] (*Zalophus californianus*). Compounds are listed in order of increasing carbon chain length. MRL = method reporting limit.

| **Compound** | **Group** | **n** | **Age-Adjusted Mean (ng/mL)** | **Standard Error** | **95% CI**  **Lower** | **95% CI**  **Upper** |
| --- | --- | --- | --- | --- | --- | --- |
| PFHxS | GSL juvenile | 25 | <MRL |  |  |  |
|  | GSL pup | 40 | <MRL |  |  |  |
|  | CSL healthy | 52 | 0.71 | 0.04 | 0.63 | 0.78 |
|  | CSL malnourished | 17 | 0.84 | 0.06 | 0.72 | 0.95 |
| PFOA | GSL juvenile | 25 | <MRL |  |  |  |
|  | GSL pup | 40 | <MRL |  |  |  |
|  | CSL healthy | 52 | 0.92 | 0.09 | 0.74 | 1.10 |
|  | CSL malnourished | 17 | 1.03 | 0.14 | 0.75 | 1.31 |
| PFOS | GSL juvenile | 25 | 2.13 | 0.80 | 0.55 | 3.71 |
|  | GSL pup | 40 | 3.79 | 0.40 | 3.01 | 4.58 |
|  | CSL healthy | 52 | 6.27 | 0.37 | 5.55 | 7.00 |
|  | CSL malnourished | 17 | 5.19 | 0.57 | 4.06 | 6.33 |
| PFNA | GSL juvenile | 25 | 0.98 | 0.51 | <MRL | 2.00 |
|  | GSL pup | 40 | 1.95 | 0.26 | 1.45 | 2.46 |
|  | CSL healthy | 52 | 2.79 | 0.24 | 2.33 | 3.26 |
|  | CSL malnourished | 17 | 3.68 | 0.37 | 2.95 | 4.40 |
| PFDA | GSL juvenile | 25 | 0.30 | 0.16 | <MRL | 0.62 |
|  | GSL pup | 40 | 0.77 | 0.08 | 0.61 | 0.93 |
|  | CSL healthy | 52 | 0.90 | 0.08 | 0.75 | 1.05 |
|  | CSL malnourished | 17 | 1.13 | 0.12 | 0.90 | 1.37 |
| PFUnA | GSL juvenile | 25 | 0.32 | 0.34 | <MRL | 0.99 |
|  | GSL pup | 40 | 1.65 | 0.17 | 1.32 | 1.98 |
|  | CSL healthy | 52 | 1.44 | 0.16 | 1.13 | 1.75 |
|  | CSL malnourished | 17 | 1.08 | 0.24 | 0.60 | 1.56 |
| Summed | GSL juvenile | 25 | 3.83 | 1.77 | 0.32 | 7.33 |
|  | GSL pup | 40 | 8.40 | 0.88 | 6.65 | 10.14 |
|  | CSL healthy | 52 | 13.11 | 0.82 | 11.49 | 14.72 |
|  | CSL malnourished | 17 | 12.96 | 1.27 | 10.45 | 15.46 |

**Supplemental Table 6.** Summary of serum PFAS concentrations in Galapagos sea lion (*Zalophus wollebaeki*) rookeries from islands in the Galapagos archipelago. Compounds are listed in order of increasing carbon chain length. *Indicates islands inhabited by humans.

| **Compound** | **Island** | **n** | **Mean (ng/mL)** | **Standard Deviation** | **Median** | **Min** | | | **Max** |
| --- | --- | --- | --- | --- | --- | --- | --- | --- | --- |
| PFOS | San Cristobal* | 25 | 2.97 | 2.19 | 2.05 | | | 0.46 | 9.37 |
|  | Santa Cruz* | 7 | 2.52 | 1.27 | 2.00 | | | 0.88 | 4.46 |
|  | Floreana* | 10 | 2.84 | 1.27 | 3.29 | | | 0.82 | 4.53 |
|  | Seymour | 10 | 2.80 | 1.48 | 2.61 | | | 0.94 | 6.42 |
|  | Rábida | 5 | 3.05 | 1.59 | 3.00 | | | 1.02 | 5.47 |
|  | Española | 3 | 7.20 | 0.57 | 7.00 | | | 6.75 | 7.84 |
|  | Marchena | 3 | 7.33 | 6.16 | 4.75 | | | 2.88 | 14.36 |
|  | Pinta | 2 | 4.56 | 0.77 | 4.56 | | | 4.01 | 5.10 |
| PFNA | San Cristobal* | 25 | 1.20 | 1.12 | 0.64 | | | <MRL | 4.70 |
|  | Santa Cruz* | 7 | 1.03 | 0.49 | 0.97 | | | <MRL | 1.79 |
|  | Floreana* | 10 | 1.21 | 0.78 | 1.25 | | | <MRL | 2.44 |
|  | Seymour | 10 | 1.47 | 1.29 | 1.11 | | | <MRL | 4.63 |
|  | Rábida | 5 | 1.85 | 1.18 | 1.78 | | | <MRL | 3.35 |
|  | Española | 3 | 4.22 | 0.35 | 4.39 | | | 3.82 | 4.44 |
|  | Marchena | 3 | 4.34 | 2.40 | 4.36 | | | 1.93 | 6.74 |
|  | Pinta | 2 | 3.14 | 0.37 | 3.14 | | | 2.88 | 3.40 |
| PFDA | San Cristobal* | 25 | 0.45 | 0.33 | 0.34 | | | <MRL | 1.46 |
|  | Santa Cruz* | 7 | 0.55 | 0.38 | 0.41 | | | <MRL | 1.13 |
|  | Floreana* | 10 | 0.45 | 0.33 | 0.27 | | | <MRL | 0.95 |
|  | Seymour | 10 | 0.54 | 0.35 | 0.46 | | <MRL | | 1.36 |
|  | Rábida | 5 | 0.68 | 0.39 | 0.67 | | <MRL | | 1.26 |
|  | Española | 3 | 1.63 | 0.09 | 1.66 | | 1.53 | | 1.70 |
|  | Marchena | 3 | 1.38 | 0.49 | 1.41 | | 0.88 | | 1.85 |
|  | Pinta | 2 | 1.46 | 0.08 | 1.46 | | 1.40 | | 1.51 |
| PFUnA | San Cristobal* | 25 | 0.84 | 0.67 | 0.68 | | <MRL | | 2.56 |
|  | Santa Cruz* | 7 | 1.56 | 1.30 | 1.38 | | <MRL | | 3.50 |
|  | Floreana* | 10 | 0.83 | 0.80 | 0.42 | | <MRL | | 2.64 |
|  | Seymour | 10 | 1.17 | 0.65 | 1.13 | | <MRL | | 2.52 |
|  | Rábida | 5 | 1.25 | 0.66 | 1.40 | | 0.30 | | 2.11 |
|  | Española | 3 | 2.76 | 0.19 | 2.78 | | 2.56 | | 2.95 |
|  | Marchena | 3 | 2.97 | 1.27 | 2.79 | | 1.80 | | 4.32 |
|  | Pinta | 2 | 2.86 | 0.09 | 2.86 | | 2.80 | | 2.92 |
| Summed | San Cristobal* | 25 | 5.57 | 4.65 | 4.71 | | 0.46 | | 19.31 |
|  | Santa Cruz* | 7 | 5.73 | 3.53 | 4.50 | | 1.96 | | 11.43 |
|  | Floreana* | 10 | 5.74 | 3.31 | 6.46 | | 1.09 | | 10.63 |
|  | Seymour | 10 | 6.00 | 3.90 | 5.61 | | 0.94 | | 15.31 |
|  | Rábida | 5 | 6.79 | 3.84 | 6.91 | | 1.46 | | 12.19 |
|  | Española | 3 | 16.12 | 0.12 | 16.09 | | 16.03 | | 16.25 |
|  | Marchena | 3 | 16.84 | 11.31 | 13.61 | | 7.49 | | 29.41 |
|  | Pinta | 2 | 12.02 | 0.57 | 12.02 | | 11.61 | | 12.42 |
